# Osteosarcoma cells with genetic signatures of BRCAness are susceptible to the PARP inhibitor talazoparib alone or in combination with chemotherapeutics

**DOI:** 10.18632/oncotarget.10720

**Published:** 2016-07-20

**Authors:** Florian Engert, Michal Kovac, Daniel Baumhoer, Michaela Nathrath, Simone Fulda

**Affiliations:** ^1^ Institute for Experimental Cancer Research in Pediatrics, Goethe-University, Frankfurt, Germany; ^2^ German Cancer Consortium (DKTK), Heidelberg, Germany; ^3^ German Cancer Research Center (DKFZ), Heidelberg, Germany; ^4^ Bone Tumour Reference Centre at the Institute of Pathology, University Hospital Basel, Basel, Switzerland; ^5^ Institute of Radiation Biology, Clinical Cooperation Group Osteosarcoma, Helmholtz Zentrum München, Neuherberg, Germany; ^6^ Pediatric Oncology Center, Department of Pediatrics, Technische Universität München and Comprehensive Cancer Center, Munich, Germany; ^7^ Department of Pediatric Hematology and Oncology, Klinikum Kassel, Kassel, Germany

**Keywords:** PARP1, PARP inhibitor, apoptosis, cell death, osteosarcoma

## Abstract

We recently discovered mutation signatures reminiscent of BRCA deficiency in the vast majority of a set of primary osteosarcomas (OS). In the current study, we therefore investigated the sensitivity of a panel of OS cell lines to the poly(ADP)-ribose polymerase (PARP) inhibitor talazoparib alone and in combination with several chemotherapeutic drugs (i.e. temozolomide (TMZ), SN-38, doxorubicin, cisplatin, methotrexate (MTX), etoposide/carboplatin). Here, we identified an association between homologous recombination (HR) repair deficiency and the response of OS cell lines to talazoparib. All OS cell lines with molecular features characteristic of *BRCA1/2* mutant tumors (so-called “BRCAness”), such as disruptive gains in *PTEN* or *FANCD2* and/or losses of *ATM, BAP1, BARD1 or CHEK2*, were susceptible to talazoparib-induced reduction of cell viability (i.e. MG63, ZK-58,, SaOS-2 and MNNG-HOS). Consistent with their high sensitivity to talazoparib, MG63 and ZK-58 cells scored positive in a DNA-based measure of genomic instability (i.e. homologous recombination deficiency (HRD)-loss of heterozygosity (LOH) score). In contrast, U2OS cells that carry a heterozygous *BRCA2* mutation and therefore most likely have one intact *BRCA2* allele left proved to be resistant to talazoparib. Furthermore, we identified TMZ as the most potent chemotherapeutic drug together with talazoparib to synergistically reduce cell viability, as confirmed by calculation of combination index (CI) values, and to suppress long-term clonogenic survival. Mechanistically, talazoparib and TMZ cooperated to induce apoptotic cell death, as demonstrated by activation of BAX and BAK, loss of mitochondrial membrane potential (MMP), caspase activation, DNA fragmentation and caspase-dependent cell death. Genetic silencing of BAX and BAK or pharmacological inhibition of caspases by zVAD.fmk significantly rescued OS cells from talazoparib/TMZ-induced apoptosis. These findings have important implications for the development of novel treatment strategies using PARP inhibitors alone or together with chemotherapy in a subset of OS with features of BRCAness.

## INTRODUCTION

OS is the most common primary bone cancer in children and young adults [1]. There is a high demand for more effective treatment options for OS patients, as the outcome of patients with refractory and/or metastatic disease remains poor with five-year survival rates below 30% [2]. Recently, we showed that the majority of OS harbor defects in HR repair either due to *BRCA1/2* mutations or to mutations in HR pathway genes, such as *FANCD2* [3]. Loss-of-function mutations in *BRCA1* or *BRCA2* as well as BRCAness have previously been reported to confer sensitivity towards PARP inhibitors, since ineffective HR repair induces a dependency on base excision repair to cope with DNA damage [4]. Thus, blockage of base excision repair using PARP inhibitors causes synthetic lethality in cancers with defects in HR repair, as cells are no longer capable to repair the cumulating DNA damage and undergo cell death [5, 6].

Currently, several PARP inhibitors (for example olaparib, talazoparib, veliparib, niraparib and rucaparib) are being investigated in clinical trials [7]. In 2014 the Food and Drug Administration (FDA) approved the first PARP inhibitor, olaparib, for the treatment of BRCA-mutated ovarian cancer and recently designated a breakthrough therapy status for olaparib in BRCA- or ATM-mutated castration-resistant prostate cancer [8, 9]. All PARP inhibitors inhibit the catalytic function of PARP1, required for PARylation of damaged DNA, while they differ in their ability to trap PARP1 to DNA, leading to replication fork stalling and cell death [10]. Talazoparib has been reported to harbor the highest PARP1 trapping activity, while its ability to inhibit catalytic PARP1 function is comparable to other inhibitors [10, 11]. Therefore, we focused our study on the PARP inhibitor talazoparib.

Programmed cell death such as apoptosis plays an important role in the response of tumors to cytotoxic therapies, as most cytotoxic therapies exert their antitumor activity by engaging programmed cell death [12]. Apoptosis can be activated by two well-defined pathways, i.e. the extrinsic (death receptor) and the intrinsic (mitochondrial) pathway, which both finally culminate in the activation of caspases as effectors of cell death [13]. Activation of the mitochondrial pathway leads to mitochondrial outer membrane permeabilization (MOMP) together with release of mitochondrial intermembrane space proteins into the cytosol triggering caspase activation and apoptosis [14]. MOMP is a process tightly regulated by various proteins, including the BCL-2 family of proteins, which can be divided into three different groups, the antiapoptotic BCL-2 proteins (e.g. BCL-2, BCL-X_L_ and MCL-1), the proapoptotic multidomain BCL-2 proteins BAK and BAX and the proapoptotic BH3-only domain proteins [15].

In the current study, we aimed i) to investigate the vulnerability of a panel of OS cell lines to the PARP inhibitor talazoparib, ii) to explore the antitumor activity of talazoparib in combination with chemotherapeutics and to identify the most potent synergistic drug combinations and ii) to elucidate the molecular mechanisms of synergy with a specific focus on cell death pathways.

## RESULTS

### Response of OS cell lines to the PARP inhibitor talazoparib is associated with their genetic signatures of BRCAness

**Figure 1:**
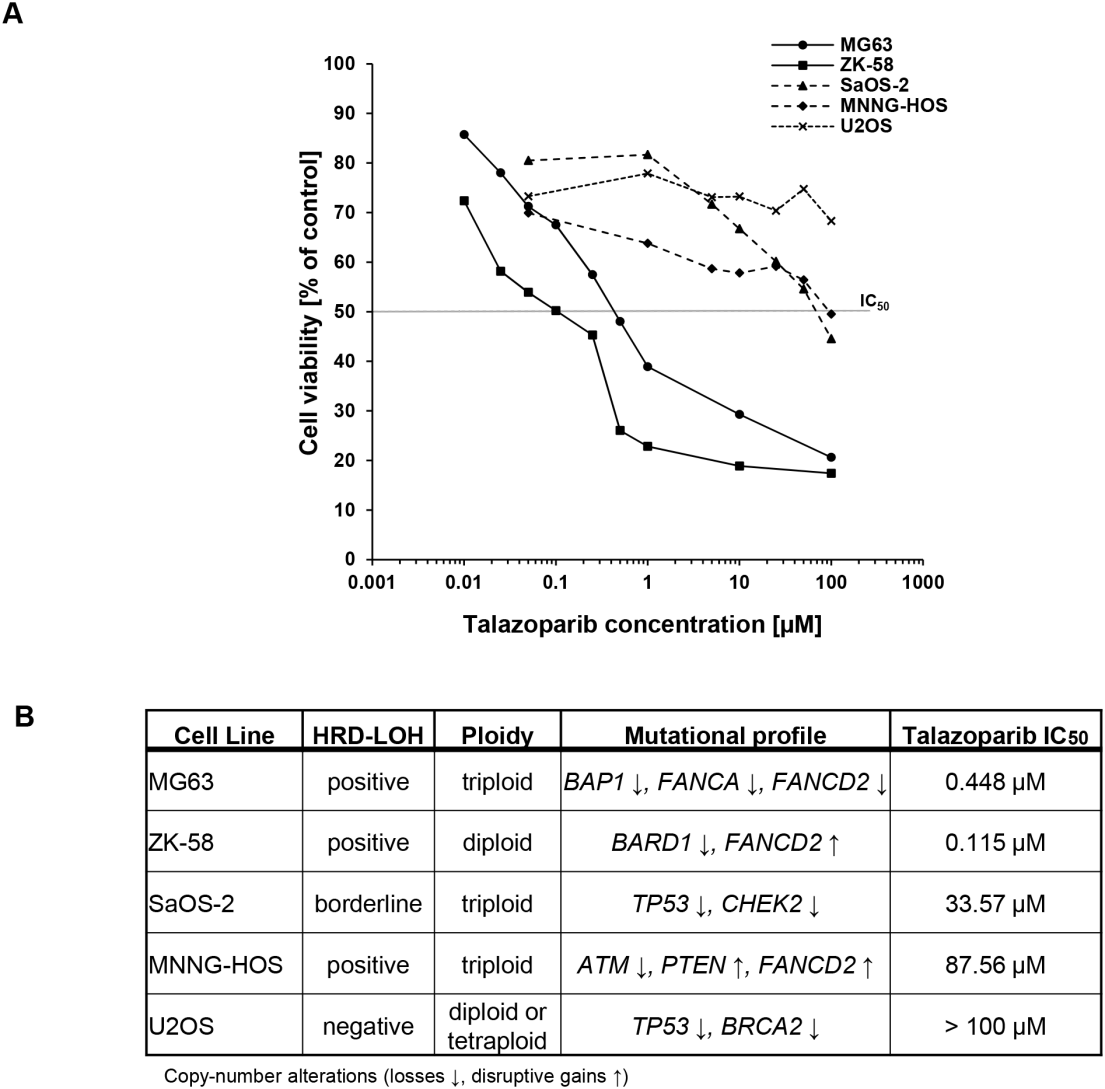
Talazoparib reduces cell viability of OS cells with genetic signatures of BRCAness **A**. OS cells were treated for 72 hours with indicated concentrations of talazoparib. Cell viability was assessed by MTT assay and is shown as percentage of untreated control. IC_50_ values were calculated using SigmaPlot™. **B**. HRD-LOH score, ploidy, mutational profile and IC_50_ values for talazoparib are indicated for five OS cell lines.

To investigate whether OS cells are susceptible to PARP1 inhibition, we tested the sensitivity of a panel of OS cell lines (i.e. MG63, ZK-58, SaOS-2, MNNG-HOS and U2OS) towards treatment with the PARP inhibitor talazoparib. According to the observed response pattern among the OS cell lines, we defined three subgroups. MG63 and ZK-58 cells displayed the highest sensitivity towards talazoparib with IC_50_ values in the nanomolar range (IC_50_: MG63 = 0.448 μM, ZK-58 = 0.115 μM) (Figure 1A). SaOS-2, MNNG-HOS cells showed intermediate sensitivity to talazoparib with micromolar IC_50_ values (IC_50_: SaOS-2 = 33.57 μM, MNNG-HOS = 87.56 μM), whereas U2OS cells remained largely resistant (≈70% viability at 100 μM talazoparib) (Figure 1A).

Generally, this response of the OS cell lines to monotherapy with talazoparib correlated with their genetic background, as OS cell lines that were susceptible to talazoparib-mediated reduction of cell viability harbor defects in HR pathway genes. MG63 cells exhibit losses in *BAP1*, *FANCA* and *FANCD2* (Figure 1B). ZK-58 cells carry a disruptive gain in *FANCD2* and loss of *BARD1*, SaOS-2 cells harbor losses in *CHEK2* and *TP53*, and MNNG-HOS cells are characterized by a loss of *ATM* as well as by disruptive gains of *PTEN* and *FANCD2* (Figure 1B). In contrast, the talazoparib-resistant cell line U2OS carries a heterozygous *BRCA2* mutation (Figure 1B), most likely with one intact *BRCA2* allele left.

To explore whether the observed response of OS cell lines to talazoparib is associated with their degree of genomic instability as a consequence of HR repair deficiency, we determined HRD-LOH score, a DNA-based measure of genomic instability [16]. Interestingly, MG63 and ZK-58 scored positive for HRD-LOH, consistent with their high sensitivity to talazoparib, whereas U2OS cells that were resistant to talazoparib scored negative for HRD-LOH (Figure 1B). SaOS-2 and MNNG-HOS cells that exhibited intermediate vulnerability to talazoparib were borderline, respectively positive by HRD-LOH score (Figure 1B). Taken together, this set of experiments indicates that the response of OS cell lines to the PARP inhibitor talazoparib is associated with their mutation signatures and the resulting degree of BRCAness.

### Screening for synergistic drug interactions of talazoparib and chemotherapeutic drugs

**Figure 2:**
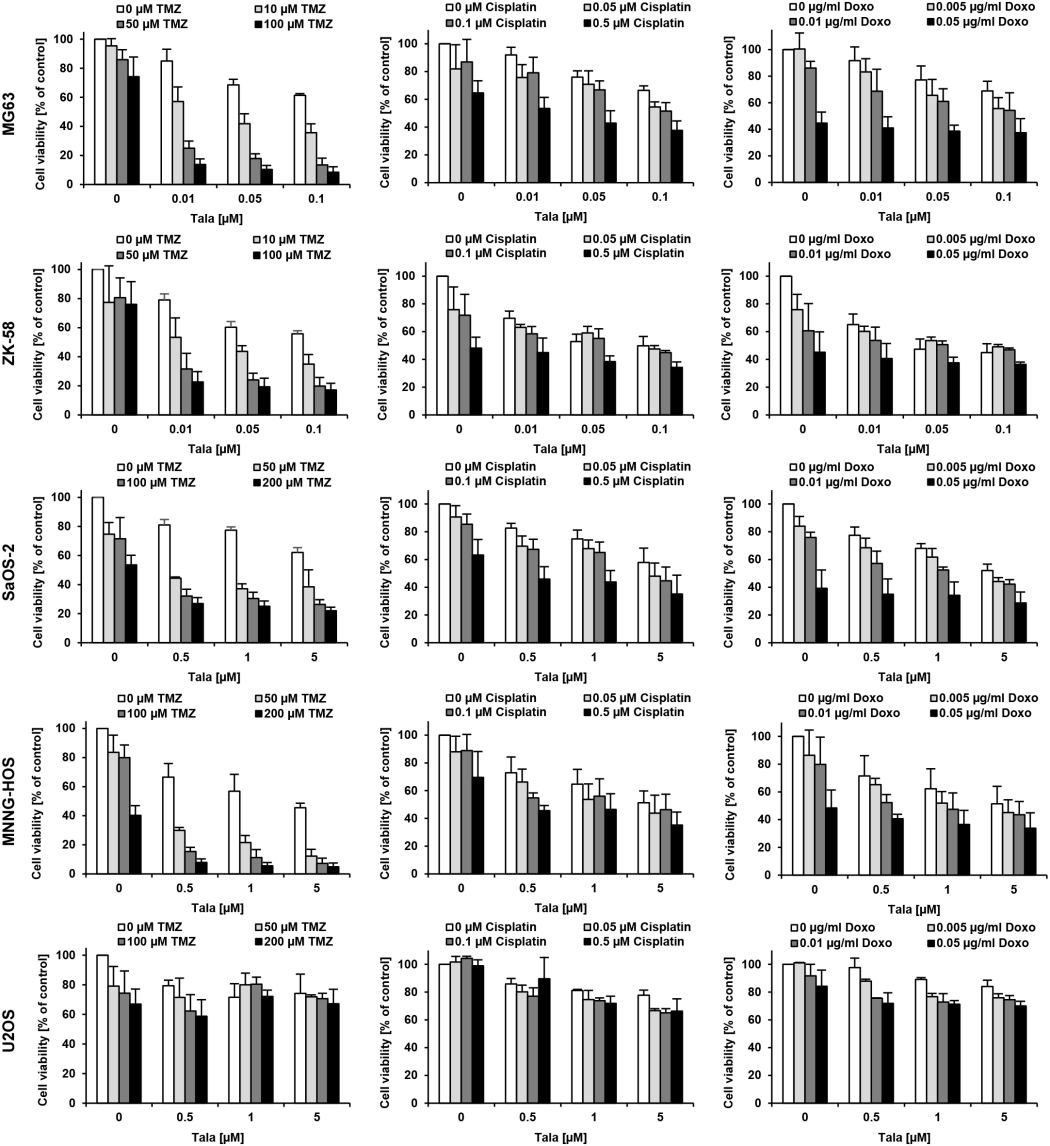
Screening for drug interactions of talazoparib and chemotherapeutic drugs in OS cells MG63, ZK-58, SaOS-2, MNNG-HOS and U2OS cells were treated for 72 hours with indicated concentrations of talazoparib in combination with indicated concentrations of TMZ, cisplatin and doxorubicin. Cell viability was assessed by MTT assay and is expressed as percentage of untreated cells. Data are shown as mean +/− SD of three independent experiments performed in triplicate.

**Figure 3:**
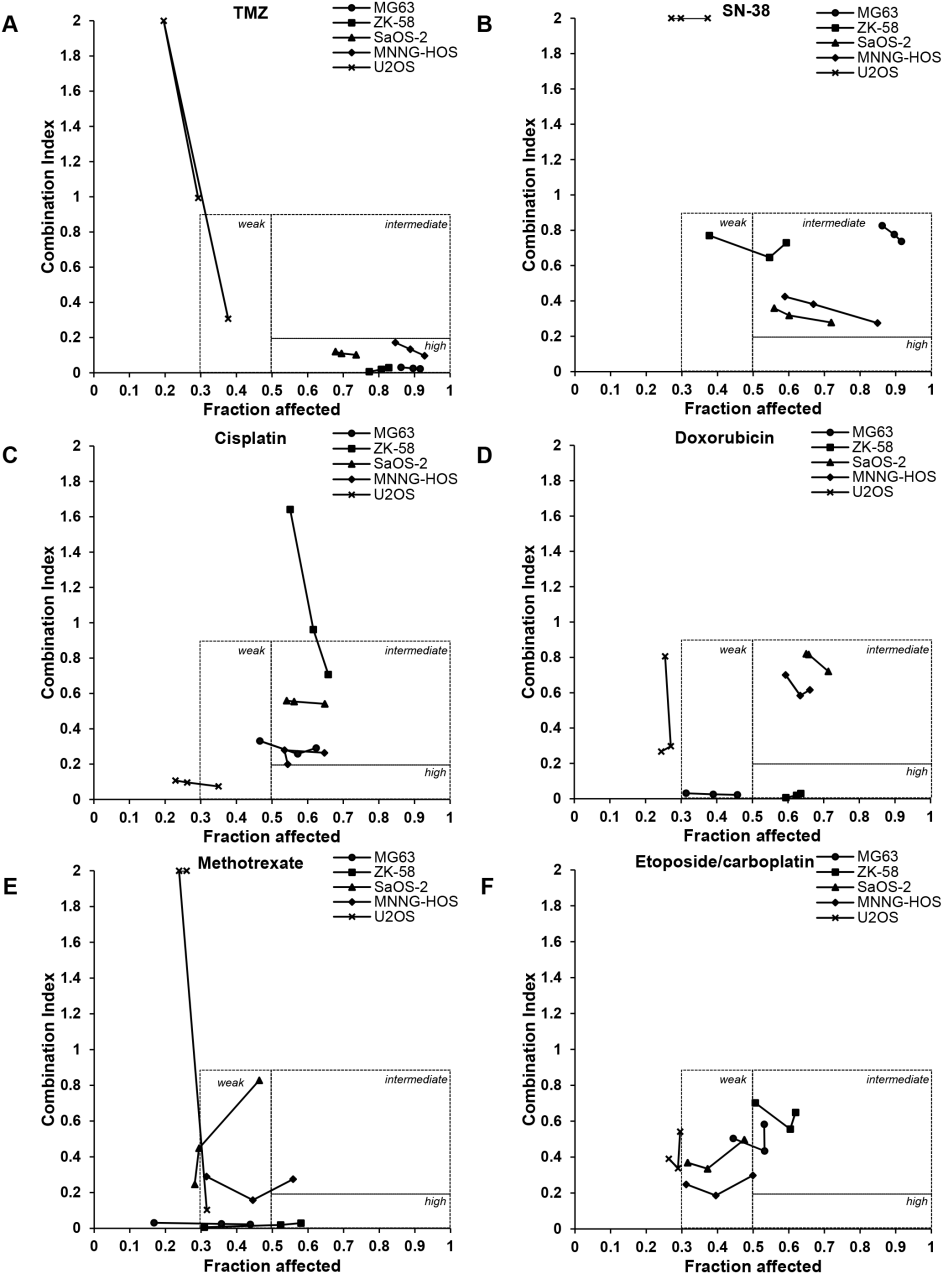
Talazoparib synergizes with several chemotherapeutic drugs, in particular with TMZ in OS cells **A-F**. CI/Fa plots were created according to the Materials and Methods section based on data shown in Figure 2 for combinations of talazoparib and chemotherapeutic drugs. CI/Fa values are shown for talazoparib concentrations ranging from 10 nM to 5 μM depending on the cell line (MG63, ZK-58: 10, 50, 100 nM; SaOS-2, MNNG-HOS, U2OS: 0.5, 1, 5 μM) in combination with the following concentrations of chemotherapeutics: 100 μM TMZ (A), 2 nM SN-38 (B), 0.5 μM cisplatin for MG63, ZK-58, SaOS-2 and MNNG-HOS and 0.1 μM cisplatin for U2OS (C), 0.01 μg/ml doxorubicin for MG63 and U2OS and 0.05 μg/ml doxorubicin for ZK-58, SaOS-2 and MNNG-HOS (D), 0.001 μg/ml MTX for MG63, 0.005 μg/ml for MNNG-HOS and U2OS and 0.01 μg/ml for ZK-58 and SaOS-2 (E), 0.1 μg/ml etoposide/carboplatin for U2OS and 0.02 μg/ml etoposide/carboplatin for MG63, ZK-58, SaOS-2 and MNNG-HOS (F).

To address the question as to whether or not PARP inhibitors modulate chemosensitivity of OS cells, we tested the effect of talazoparib in combination with several established anticancer drugs, such as TMZ, cisplatin, doxorubicin, SN-38, MTX and etoposide/carboplatin (Figure 2 and Supplementary Figure 1). We used suboptimal concentrations of talazoparib and anticancer drugs that caused up to 20-40% reduction of cell viability when used as monotherapy compared to solvent-treated controls. To identify synergistic drug interactions we calculated CI values and fraction affected (Fa) values [17], which are shown as CI over Fa plots for each drug (Figure 3, Supplementary Table 1). For comparison, we selected drug combinations that lead to the most pronounced synergy (lowest CI values and highest Fa values), full information on CI and Fa values are shown in Supplementary Table 1.

This analysis revealed three different classes of synergy of chemotherapeutics with talazoparib (10 nM) in four of the tested OS cell lines (i.e. MG63, ZK-58, SaOS-2 and MNNG-HOS) that we defined as highly synergistic (CI <0.2, Fa >0.5), intermediately synergistic (CI >0.2<0.9, Fa >0.5) and weakly synergistic (CI >0.2<0.9, Fa >0.3<0.5). In contrast, we found no consistent synergistic interaction of talazoparib with the tested anticancer drugs in U2OS cells, as CI values showed no synergism (CI >0.9) and/or Fa values were below 0.3 with the exception of a few drug combinations (Figure 3, Supplementary Table 1).

For TMZ in combination with talazoparib we detected a highly synergistic drug interaction in all four responding OS cell lines (i.e. MG63, ZK-58, SaOS-2 and MNNG-HOS), as indicated by CI values < 0.2 and Fa values > 0.7 (Figure 3A, Supplementary Table 1). For SN-38 together with talazoparib we observed intermediate synergy with some variations between the responding four OS cell lines. SaOS-2 and MNNG-HOS cells proved to be more responsive to cotreatment with talazoparib and SN-38 (CI < 0.4 and Fa > 0.5) compared to MG63 and ZK-58 cells (CI ≈ 0.8 and Fa > 0.5) (Figure 3B, Supplementary Table 1). Intermediate synergy was also found for the combination of talazoparib and cisplatin or doxorubicin (Figure 3C, 3D, Supplementary Table 1). However, the response pattern to the cotreatment with doxorubicin and talazoparib was more heterogeneous, as SaOS-2 and MNNG-HOS cells displayed intermediate synergism, ZK-58 cells high synergism and MG63 cells weak synergism (Figure 3D). Weak synergy was predominately found for the combination of MTX with talazoparib, as only selected drug combinations yielded synergistic CI values in more than 50% of the cell population in ZK-58 and MNNG-HOS cells (Figure 3E, Supplementary Table 1). A weak to intermediate synergism depending on the cell line was detected for the combination of etoposide/carboplatin and talazoparib (Figure 3F, Supplementary Table 1). Thus, TMZ turned out to be the most potent and promising anticancer drug that consistently acted in concert with talazoparib to reduce cell viability in a highly synergistic manner in four OS cells.

### Talazoparib/TMZ cotreatment is superior to cisplatin or doxorubicin to trigger cell death and to suppress clonogenic growth

**Figure 4:**
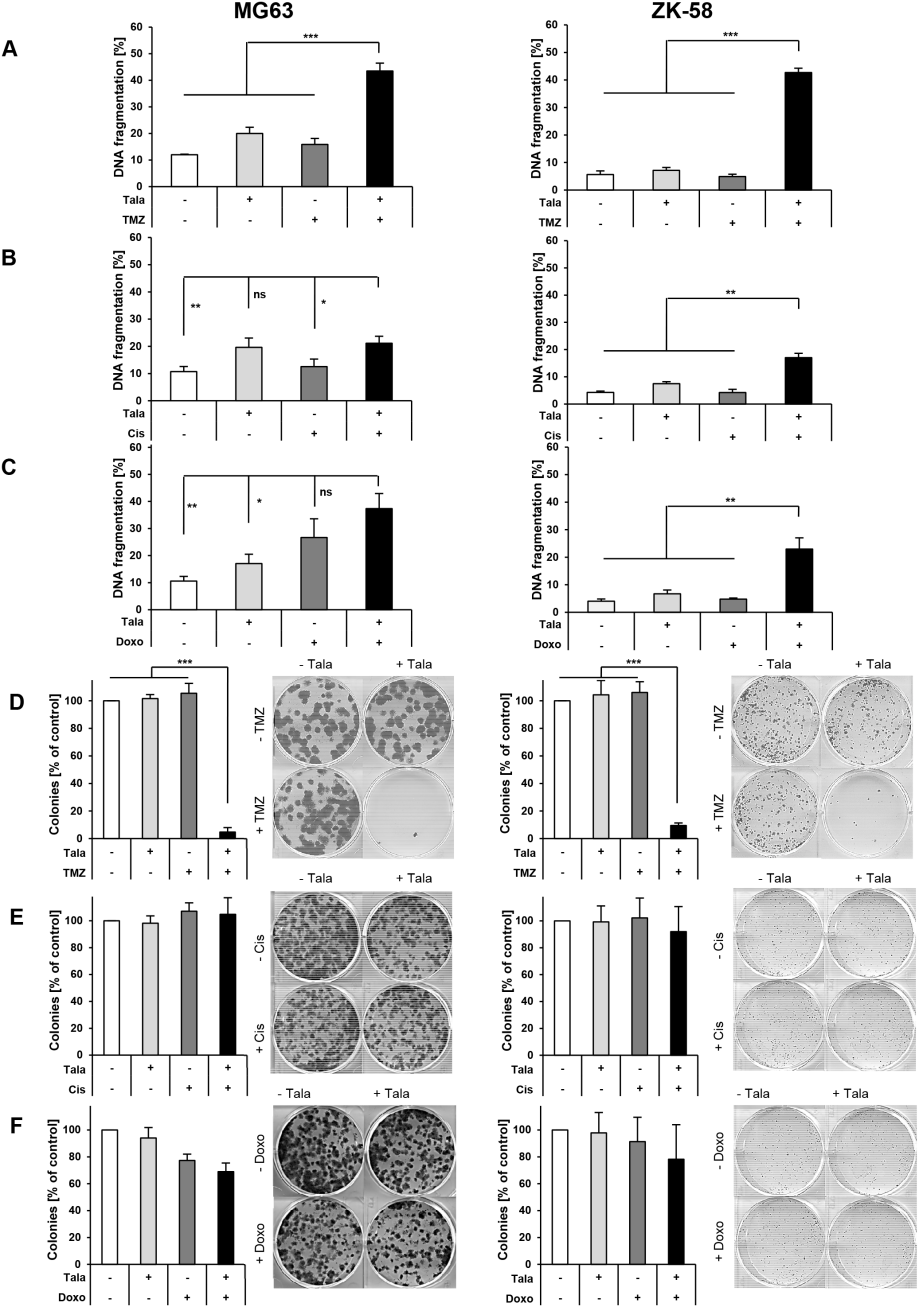
Talazoparib/TMZ cotreatment is superior to cisplatin or doxorubicin to trigger cell death and to suppress clonogenic growth **A-C**. MG63 and ZK-58 cells were treated for 72 hours with 10 nM talazoparib and/or 100 μM TMZ (A), 50 nM cisplatin (B) or 10 ng/ml doxorubicin (C). Apoptosis was determined by quantification of DNA fragmentation of PI-stained nuclei using flow cytometry. Data are shown as mean +/− SD of three independent experiments performed in triplicate; *, P < 0.05; **, P < 0.01; ***, P < 0.001; ns, not significant. **D-F**. MG63 and ZK-58 cells were treated with 10 nM talazoparib and/or 100 μM TMZ (D), 50 nM cisplatin (E) or 10 ng/ml doxorubicin (F) for 24 hours, living cells were counted and subsequently 100 cells/well were re-seeded in drug-free medium in a six-well plate. Colony formation was assessed after 12 days for talazoparib/TMZ cotreatment (D) and after 10 days for talazoparib/cisplatin and talazoparib/doxorubicin cotreatment (E, F) by crystal violet staining and colonies were counted macroscopically. The number of colonies is expressed as percentage of untreated control and representative images are shown. Data are shown as mean +/− SD of three independent experiments performed in triplicate; ***, P < 0.001.

Since we identified MG63 and ZK-58 cells to be most sensitive to treatment with talazoparib alone (Figure 1A) and in combination with TMZ (Figures 2, 3A), we selected those cell lines for our subsequent studies. To further assess the antitumor activity of selected drug combinations we performed additional assays and determined DNA fragmentation as a marker of apoptotic cell death and colony formation as a parameter of long-term clonogenic survival. For these talazoparib-based combinations, we focused on TMZ, since it showed the most pronounced synergy together with talazoparib, and on cisplatin and doxorubicin, as they are generally used in the standard treatment of OS [2]. Notably, talazoparib cooperated with TMZ to significantly increase DNA fragmentation and to suppress colony formation in both OS cell lines (Figure 4A, 4D). While talazoparib acted in concert with cisplatin or doxorubicin to significantly enhance DNA fragmentation in ZK-58 cells, these combinations were not more effective than single agents alone to cause DNA fragmentation in MG63 cells (Figure 4B, 4C). In addition, cotreatment with talazoparib and cisplatin or doxorubicin failed in both OS cell lines to inhibit colony formation significantly better than treatment with these drugs alone (Figure 4E, 4F). Together, these findings suggest that the combination of talazoparib with TMZ is superior to the combination of talazoparib with cisplatin or doxorubicin to trigger cell death and to suppress long-term clonogenic survival in the two investigated OS cell lines. However, further investigation in additional OS cell lines is required to address the question as to whether or not talazoparib is suitable to increase chemosensitivity of OS cells to cisplatin or doxorubicin. In the following, we focused our mechanistic studies on the combination of talazoparib and TMZ.

### Talazoparib/TMZ cotreatment causes caspase-dependent cell death

**Figure 5:**
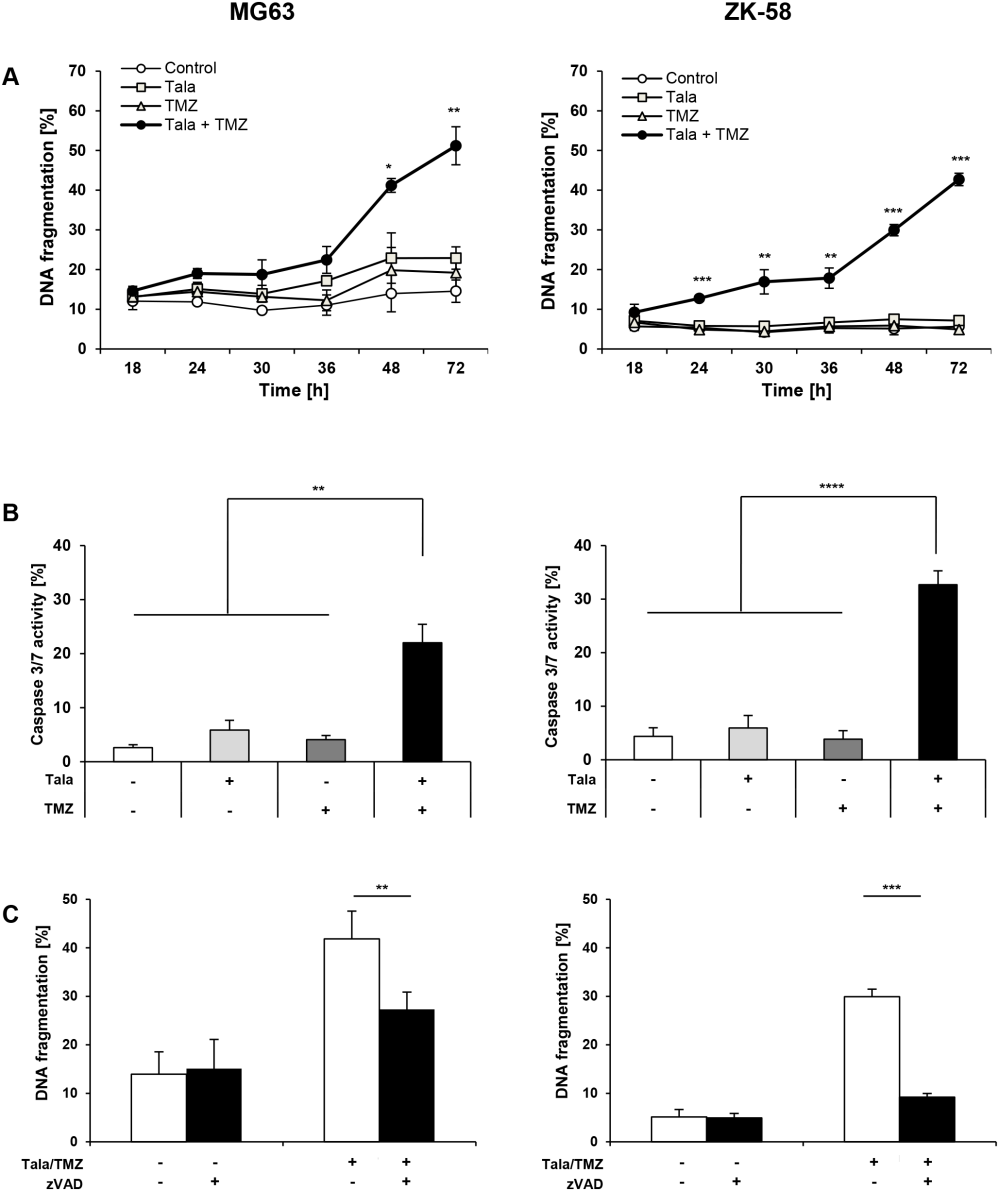
Talazoparib/TMZ cotreatment causes caspase-dependent cell death **A**. MG63 and ZK-58 cells were treated with 10 nM talazoparib and/or 100 μM TMZ for indicated times. Apoptosis was determined by quantification of DNA fragmentation of PI-stained nuclei using flow cytometry. Data are shown as mean +/− SD of three independent experiments performed in triplicate; *, P < 0.05; **, P < 0.01; ***, P < 0.001. **B**. MG63 and ZK-58 cells were treated with 10 nM talazoparib and/or 100 μM TMZ for 48 hours. Cell nuclei were stained with Hoechst 33342 and caspase activity was measured using caspase-3/7 cell reagent and microscopy. Data are shown as mean +/− SD of three independent experiments performed in triplicate; **, P < 0.01; ***, P < 0.001. **C**. MG63 and ZK-58 cells were treated with 10 nM talazoparib and 100 μM TMZ for 48 hours in the presence or absence of 50 μM zVAD.fmk. Apoptosis was determined by quantification of DNA fragmentation of PI-stained nuclei using flow cytometry. Data are shown as mean +/− SD of three independent experiments performed in triplicate; **, P < 0.01; ***, P < 0.001.

To better understand the molecular mechanisms of cell death underlying the synergistic antitumor activity of talazoparib/TMZ combination treatment, we monitored the kinetics of cell death. Talazoparib acted in concert with TMZ to increase DNA fragmentation in a time-dependent manner in both cell lines with a significant increase in cell death upon talazoparib/TMZ cotreatment at 24 hours in ZK-58 cells and at 48 hours in MG63 cells (Figure 5A). To monitor activation of caspases as typical effector molecules of apoptosis we performed a caspase-3/7 activity assay. Talazoparib cooperated with TMZ to significantly increase caspase-3/7 activity in both cell lines (Figure 5B). To investigate whether caspase activity is required for the induction of apoptosis, we used the broad-range caspase inhibitor zVAD.fmk, which significantly decreased talazoparib/TMZ-induced apoptosis in both cell lines (Figure 5C). However, the protection by zVAD.fmk was only partial in MG63 cells (Figure 5C), suggesting that caspase-independent mechanisms might also be involved. This set of experiments demonstrates that talazoparib/TMZ-induced cell death is time-dependent with slower kinetics in MG63 than ZK-58 cells and occurs in a caspase-dependent manner.

To explore whether the addition of talazoparib would potentiate the cytotoxicity of TMZ also in non-malignant cells, we tested the effect of TMZ in the presence and absence of talazoparib on human peripheral blood lymphocytes (PBLs) that were freshly isolated from two different donors. We used PBLs, as hematological toxicity has been described for talazoparib [18]. Neither single treatment of talazoparib or TMZ nor the combination of talazoparib/TMZ caused any detectable cytotoxicity in PBLs compared to untreated controls (Supplementary Figure 2) at the same time point and at similar concentrations that cooperated to trigger cell death in OS cells (Figure 4A). These results point to some tumor selectivity of the talazoparib/TMZ combination treatment.

### Talazoparib/TMZ cotreatment triggers BAX/BAK activation and MOMP

**Figure 6:**
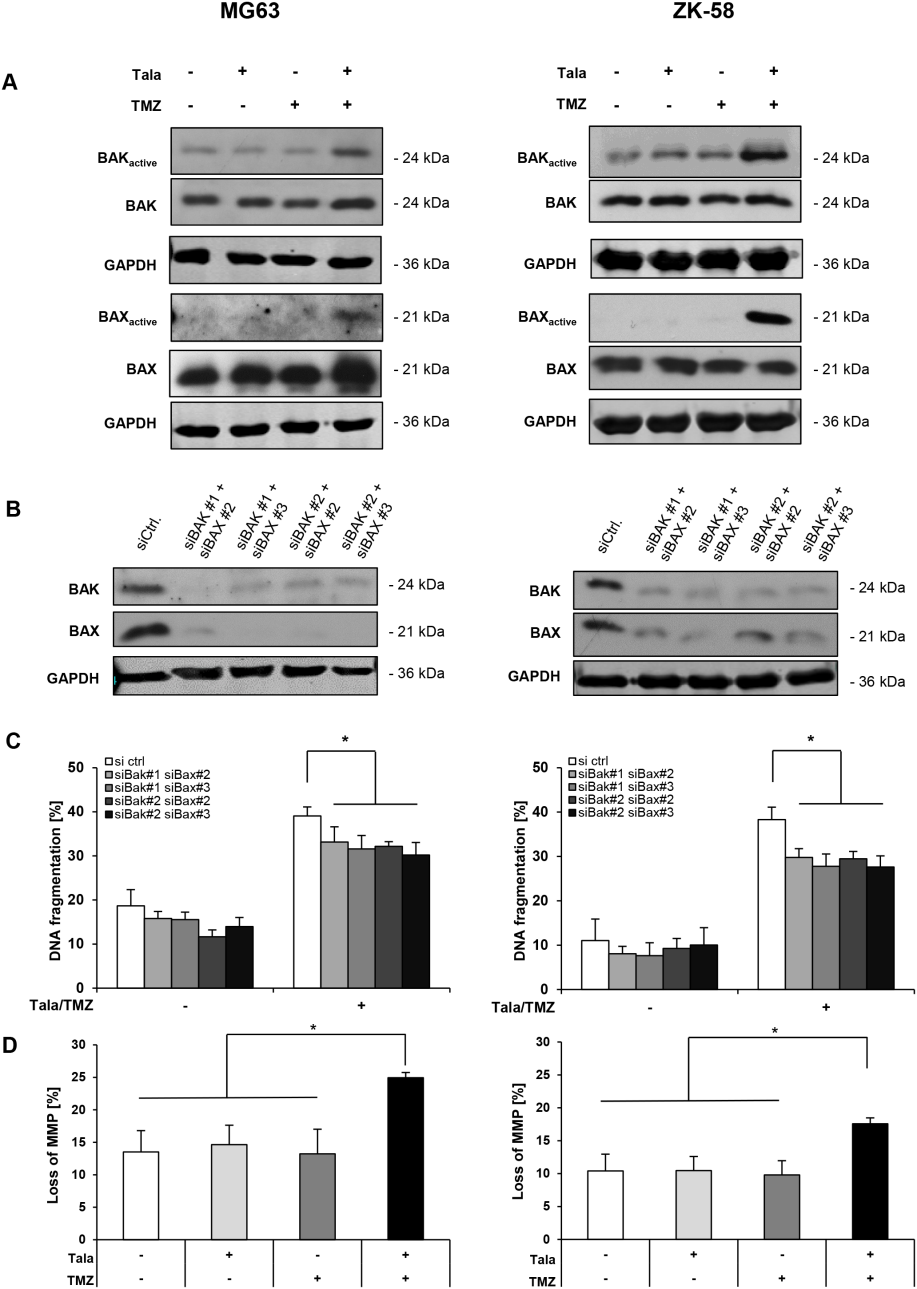
Talazoparib/TMZ cotreatment triggers BAX/BAK activation and MOMP **A**. MG63 and ZK-58 cells were treated with 10 nM talazoparib and/or 100 μM TMZ for 36 hours (MG63) or 24 hours (ZK-58). Active conformations of BAK or BAX were immunoprecipitated using active conformation-specific antibodies and were analyzed by Western blotting. Expression of total BAK or BAX and GAPDH served as loading controls. Representative blots of two independent experiments are shown. **B** and **C**. MG63 and ZK-58 cells were transiently transfected with 10 nM non-silencing siRNA or 5 nM each of different combinations of constructs targeting BAK or BAX and expression of BAK and BAX was analyzed by Western blotting, GAPDH served as loading control (B). Representative blots of two independent experiments are shown. Transiently transfected OS cells were treated for 48 hours with 10 nM talazoparib and 100 μM TMZ and apoptosis was determined by quantification of DNA fragmentation of PI-stained nuclei using flow cytometry (C). Data are shown as mean +/− SD of three independent experiments performed in triplicate; *, P < 0.05. **D**. MG63 and ZK-58 cells were treated with 10 nM talazoparib and/or 100 μM TMZ for 36 hours (MG63) or 24 hours (ZK-58). Loss of MMP in the living cell population was determined by flow cytometry using JC-1 fluorescent dye. Data are shown as mean +/− SD of three independent experiments performed in triplicate; *, P < 0.05.

Next, we asked whether talazoparib and TMZ cooperate to activate BAK and BAX, two multidomain proapoptotic BCL-2 proteins that control MOMP [15]. To address this question, we immunoprecipitated BAK and BAX using conformation-specific antibodies, since activation of BAK and BAX is accompanied by a conformational change. Strikingly, talazoparib and TMZ acted in concert to activate BAK and BAX in both OS cell lines compared to treatment with either agent alone (Figure 6A). We detected BAK and BAX activation upon talazoparib/TMZ treatment at a later time point (i.e. 36 hours) in MG63 cells compared to ZK-58 cells (i.e. 24 hours) (Figure 6A), consistent with the slower kinetics of cell death in MG63 compared to ZK-58 cells (Figure 5A). To explore whether BAK and BAX activation is required for talazoparib/TMZ-induced apoptosis, we concomitantly silenced BAK and BAX by siRNAs (Figure 6B). Notably, knockdown of BAK and BAX significantly rescued talazoparib/TMZ-mediated apoptosis in all different combinations of siRNA constructs used to silence BAK and BAX (Figure 6C). However, BAK/BAX silencing only partly, yet significantly reduced talazoparib/TMZ-induced apoptosis (Figure 6C), pointing also to BAK/BAX-independent mechanisms. Since activation of BAK and BAX leads to pore formation in the outer mitochondrial membrane accompanied by loss of MMP, we then assessed the MMP by JC-1 staining, a dye that accumulates in polarized mitochondria. Talazoparib and TMZ cooperated to trigger a significant loss of MMP in both cell lines (Figure 6D). These findings demonstrate that talazoparib/TMZ cotreatment promotes BAX/BAK activation and loss of MMP in OS cells.

## DISCUSSION

Using exome sequencing, we recently identified mutation signatures characteristic of BRCA deficiency in OS [3], indicating that OS might be susceptible to PARP inhibitors. Therefore, the current study aimed to investigate the sensitivity of a panel of OS cell lines to the PARP inhibitor talazoparib alone or in combination with various chemotherapeutics, most of them commonly used in the treatment of OS. Here, we discovered that the response of OS cell lines to monotherapy with talazoparib correlated with their mutation signatures and the resulting degree of BRCAness. All four OS cell lines that were susceptible to talazoparib-mediated inhibition of cell viability harbored genetic alterations in genes that are functionally analogous to *BRCA1/2* mutations, including disruptive gains in *PTEN* and *FANCD2* and/or losses of *ATM, BAP1, BARD1, FANCA or CHEK2*, although none of the tested OS cell lines showed a bi-allelic *BRCA1/2* mutation [3]. In contrast, talazoparib-resistant U2OS cells carry a heterozygous *BRCA2* mutation [3] and most likely have one intact *BRCA2* allele left.

Importantly, the susceptibility of OS cell lines to talazoparib broadly correlated with their degree of genomic instability, as MG63 and ZK-58 cells that exhibited high sensitivity to talazoparib with nanomolar IC_50_ values scored positive in HRD-LOH score, a DNA-based measure of genomic instability [16]. In contrast, U2OS cells that were resistant to talazoparib were HRD-LOH score-negative. Cell lines exhibiting micromolar IC_50_ values for talazoparib (i.e. SaOS-2 and MNNG-HOS cells) were borderline, respectively positive by HRD-LOH score. Testing of talazoparib monotherapy in the pediatric preclinical testing program has previously shown minimal *in vivo* activity against OS; however, the BRCAness status of OS was not reported in that study [19].

*PTEN*, *ATM*, *BAP1, BARD1*, *CHEK2*, *FANCA* and *FANCD2* have been shown to exert pivotal functions in HR repair [20]. Loss of *PTEN* function has previously been described to cause genomic instability [21], and *PTEN*-deficient cells were found to be sensitive to the PARP inhibitor olaparib [22]. Also, *ATM*-deficient lymphoid tumor cells have shown vulnerability to olaparib *in vitro* and *in vivo* [23]. Furthermore, BARD1 has been reported to be one of the main binding partners of BRCA1 and BRCA1/BARD1 heterodimer binds to DNA damage sites [24]. CHEK2-mediated phosphorylation of BRCA1 at serine 988 has been shown to regulate DNA damage response [25]. Ubiquitination of FANCD2 plays an important role in the disposal of DNA interstrand cross-links [26] and the deficiency of FANCD2, ATM or CHK2 was described to lead to non-functional HR and sensitivity to PARP inhibition [27]. Thus, loss of function in genes involved in HR repair can cause BRCAness, resulting in increased vulnerability to PARP1 inhibition.

Furthermore, among the tested chemotherapeutic drugs we identified TMZ to be most potent for combination treatment with talazoparib in OS, which was confirmed by highly synergistic CI values and by a large fraction of cells affected. In addition, talazoparib and TMZ acted together not only to reduce cell viability, but also to trigger cell death and to suppress long-term clonogenic survival. We and other groups previously demonstrated that the DNA-damaging agent TMZ is particularly suitable for PARP inhibitor-based combination treatment, yielding highly synergistic drug interaction in Ewing sarcoma cells [28–30]. Depending on the type of DNA lesions generated by genotoxic drugs, PARP1 is bound to DNA and/or required for DNA repair [28]. Methylated bases formed as a result of treatment with TMZ are repaired by base excision repair, thereby producing a repair intermediate to which PARP1 is recruited [7, 29]. Synergistic interaction of the PARP inhibitor olaparib with topoisomerase I inhibitors, doxorubicin or platinum drugs has been reported in a panel of pediatric solid tumor cell lines including two OS cell lines, while TMZ was not included in that study [31].

Our molecular studies revealed that the synergistic cytotoxicity of talazoparib and TMZ in OS cells involves the induction of apoptotic cell death. First, we showed that talazoparib/TMZ cotreatment triggered DNA fragmentation and loss of MMP as characteristic markers of apoptotic cell death. Second, talazoparib/TMZ-stimulated activation of BAK and BAX contributed to the induction of apoptosis, since genetic silencing of BAK and BAX significantly reduced talazoparib/TMZ-induced cell death. Third, talazoparib and TMZ acted together to activate caspases, which were required for apoptosis, as the caspase inhibitor zVAD.fmk significantly rescued talazoparib/TMZ-mediated cell death. While our data showing that zVAD.fmk as well as BAK/BAX silencing significantly reduced talazoparib/TMZ-induced cell death demonstrated that caspases and BAK/BAX activation contributed to talazoparib/TMZ-induced apoptosis, additional mechanisms are also likely to be involved, since the protection by zVAD.fmk or BAK/BAX knockdown was only partial.

PARP inhibitors are widely used in clinical trials for several cancers carrying *BRCA1/2* mutations or the signature of BRCAness such as ovarian cancer, prostate cancer, pancreatic cancer, glioblastoma, and soft-tissue sarcomas [32], and olaparib gained FDA approval for *BRCA1/2*-mutated ovarian cancer and castration-resistant prostate cancer [8, 9]. Together with our recent report showing that the vast majority of a set of primary OS specimens exhibit genetic traits of *BRCA* deficiency [3], our present study indicates that PARP inhibitor-based therapies may also present a novel targeted treatment option for a defined subgroup of OS patients that harbor features of BRCAness. Further studies are therefore warranted in future to extend our investigation of talazoparib/TMZ combination therapy to a larger panel of OS cell lines including primary human OS cultures and to an *in vivo* model of OS. Since the addition of talazoparib did not increase the cytotoxicity of TMZ on non-malignant PBLs, there might be a therapeutic window for the incorporation of talazoparib into treatment protocols for OS. Selective synthetic lethality of the talazoparib/TMZ combination treatment in OS cells might be explained by the fact that cancer cells but not normal cells harbor HR repair deficiency. The doses of talazoparib and TMZ that caused synergistic induction of cell death in our study correspond to clinically achievable plasma concentrations of those drugs (up to 67 nM for talazoparib [18] and up to 100 μM for TMZ [33]), underlining the potential clinical relevance of our study. Taken together, our study has important implications for the development of novel treatment strategies using PARP inhibitors alone or together with chemotherapy in a subset of OS with features of BRCAness.

## MATERIALS AND METHODS

### Cell culture and chemicals

OS cell lines (MG63, ZK-58, SaOS-2, MNNG-HOS and U2OS) were kindly provided by M. Nathrath (München, Germany) and authenticated by STR profiles. Cells were maintained in RPMI 1640 medium (Life Technologies, Inc., Darmstadt, Germany), supplemented with 10% fetal calf serum (FCS), 1% penicillin/streptomycin, 1 mM sodium pyruvate (all from Life Technologies, Inc.). Cell lines were regularly tested for mycoplasma contamination to ensure that experiments were performed only with mycoplasma-free cell lines. Talazoparib was obtained from Selleckchem (Munich, Germany). TMZ, SN-38, cisplatin, doxorubicin and etoposide were purchased from Sigma-Aldrich (Taufkirchen, Germany), MTX and carboplatin were purchased from Medac (Hamburg, Germany), zVAD.fmk from Bachem (Heidelberg, Germany). Chemicals were purchased from Sigma-Aldrich or Carl Roth (Karlsruhe, Germany) unless otherwise indicated.

### Determination of apoptosis, cell viability and colony formation

Apoptosis was determined by flow cytometric analysis (FACSCanto II, BD Biosciences, Heidelberg, Germany) of DNA fragmentation of propidium iodide (PI)-stained nuclei as described previously [34]. Cell viability was assessed by 3-(4,5-dimethylthiazol-2-yl)-2,5-diphenyltetrazolium bromide (MTT) assay according to the manufacturer's instructions (Roche Diagnostics, Mannheim, Germany). Results are expressed as percentage of cell viability relative to untreated controls. For colony formation assay cells were treated as indicated for 24 hours. Subsequently, living cells were counted, 100 cells were reseeded and cultured in drug-free medium for additional 10, respectively 12 days before fixation and staining with 0.5% crystal violet, 30% ethanol and 3% formaldehyde. Colonies were counted macroscopically.

### Western blot analysis

Western blot analysis was performed as described previously [34] using the following antibodies: BAK (BD, New Jersey, USA), BAX NT (Millipore, Darmstadt, Germany), GAPDH (HyTest, Turku, Finland). Goat anti-mouse IgG and goat anti-rabbit IgG conjugated to horseradish peroxidase (Santa Cruz Biotechnology, Heidelberg, Germany) were used as secondary antibodies and enhanced chemiluminescence (Amersham Bioscience, Freiburg, Germany) or infrared dye-labeled secondary antibodies and infrared imaging were used for detection (Odyssey Imaging System, LI-COR Bioscience, Bad Homburg, Germany). Representative blots of at least two independent experiments are shown.

### Caspase activity assay

For determining caspase activity cells were treated as indicated and caspase activity was assessed by caspase-3/7 staining according to the manufacturer's instructions for CellEvent Caspase-3/7 Green Detection Reagent (ThermoFisher scientific, Schwerte, Germany) using ImageXpress Micro XLS system (Molecular Devices, Biberach an der Riss, Germany).

### RNA interference

Cells were reversely transfected with a combination of targeting siRNAs (10nM for control siRNA, 4390844, 5 nM for BAK, s1880 and s1881; 5 nM for BAX, s1889 and s1890) using Lipofectamine RNAiMAX reagent (Life Technologies, Inc., Darmstadt, Germany) and Opti-MEM medium (Life Technologies, Inc.). After six hours of incubation with transfection solution, the medium was changed and cells recovered for 48 hours before drug treatment.

### Determination of BAK and BAX activation or MMP

BAK and BAX activation was determined by immunoprecipitation of active conformation by specific antibodies. Briefly, cells were lysed in CHAPS lysis buffer (10 mmol/l HEPES, pH 7.4; 150 mmol/l NaCl; 1% CHAPS). 500 μg protein was incubated overnight at 4°C with 8 μg mouse anti-BAX antibody (clone 6A7; Sigma) or 0.5 μg mouse anti-BAK antibody (AB-1; Calbiochem, Darmstadt, Germany) and 10 μl pan mouse IgG Dynabeads (Dako, Hamburg, Germany), washed with CHAPS lysis buffer and analyzed by Western blotting using rabbit anti-BAX NT antibody (Millipore, Darmstadt, Germany) or rabbit anti-BAK antibody (BD Biosciences). Loss of MMP was assessed by JC-1 staining according to the manufacturer's instructions (ThermoFisher scientific).

### PBL isolation

For PBL isolation, blood was drawn from two independent donors and leukocytes were isolated using density centrifugation. Afterwards, leukocytes were treated with indicated concentrations of talazoparib and/or TMZ and cell death was assessed directly after PI staining as described previously [34].

### HRD-LOH analysis

Affymetrix CytoscanHD array data processed with Nexus 8.0 software such that only segments with copy-number changes larger than 100 Kb and with at least 21 probes were considered for analysis using a public algorithm. In brief, HRD-LOH score requires at least 15 sub-chromosomal LOH segments of a size exceeding 15 Mb, which do not affect the whole chromosome and are at least 10 Mb apart. Borderline cases were tumors with 14 LOH events, while negative cases had 13 or less LOH events.

### Statistical analysis

Statistical significance was assessed by Student's t-Test (two-tailed distribution, two-sample, equal variance) using Microsoft Excel (Microsoft Deutschland GmbH, UnterschleiΔheim, Germany). Drug interactions by CI values and Fa were analyzed by the Chou-Talalay method described by Chou [17, 35] using CalcuSyn software (Biosoft, Cambridge, UK).

## SUPPLEMENTARY DATA FIGURES AND TABLE




