# Supplementary material for: Osteosarcoma cells with genetic signatures of BRCAness are susceptible to the PARP inhibitor talazoparib alone or in combination with chemotherapeutics
Source: Oncotarget. 2016 Jul 20;8(30):48794–806. doi: 10.18632/oncotarget.10720 (PMC5564725; doi:10.18632/oncotarget.10720)
Supplement: Supplementary file 1 [file oncotarget-08-48794-s001.pdf]

Osteosarcoma cells with genetic signatures of BRCAness are susceptible to the PARP inhibitor talazoparib alone or in combination with chemotherapeutics

SUPPLEMENTARY DATA

Cell death of non-adherent cells was determined by PI staining and flow cytometry to determine plasma membrane integrity as described previously [1].

REFERENCES

1. Heinicke U, Kupka J, Fichter I, Fulda S. Critical role of mitochondria-mediated apoptosis for JNJ-26481585-induced antitumor activity in rhabdomyosarcoma. Oncogene. 2015; Nov 30 [E-pub ahead of print].

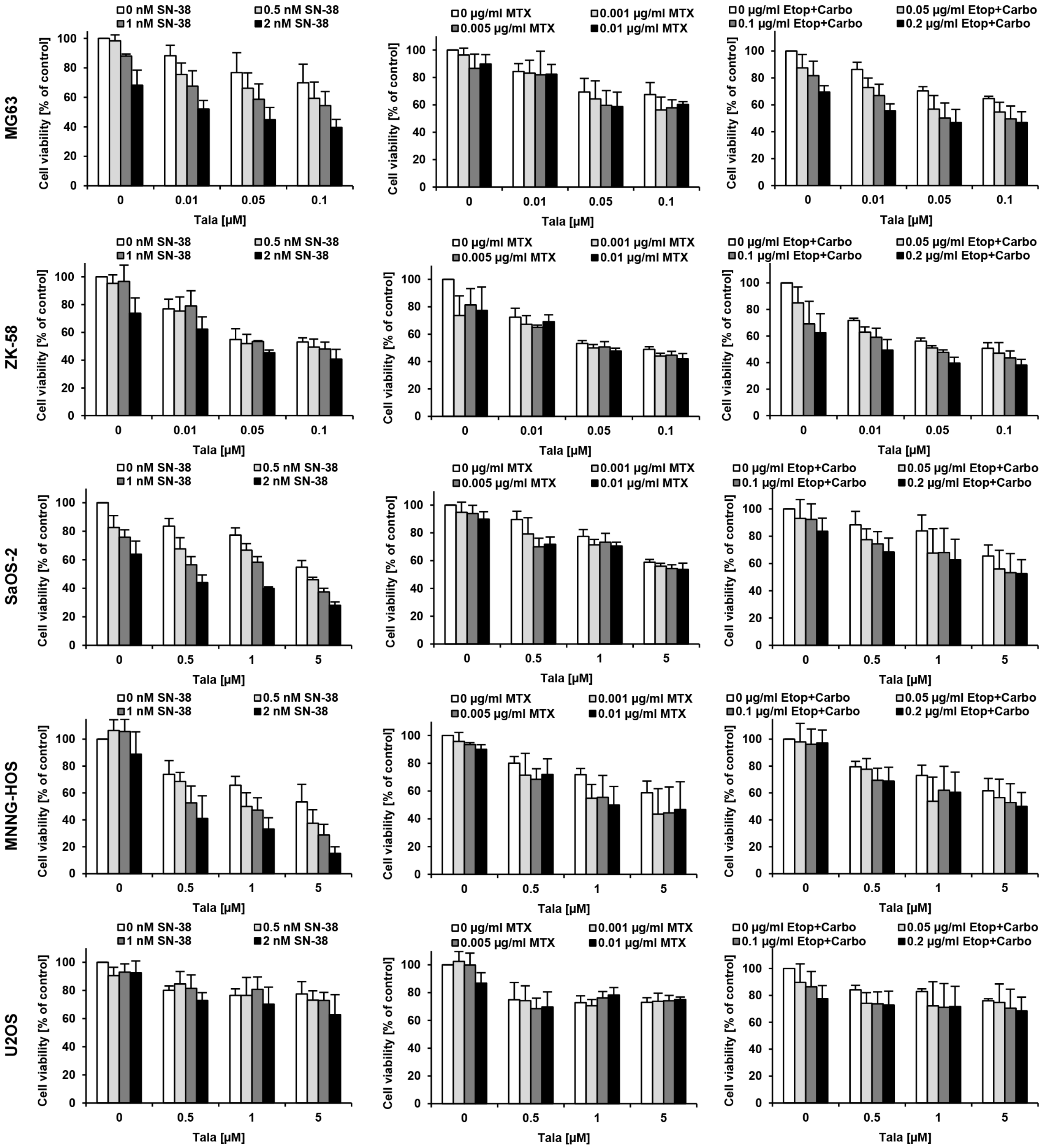

Supplementary Figure S1: Screening for drug interactions of talazoparib and chemotherapeutic drugs in OS cells. MG63, ZK-58, SaOS-2, MNNG-HOS and U2OS cells were treated for 72 hours with indicated concentrations of talazoparib in combination with indicated concentrations of the anticancer drugs SN-38, MTX and a combination of etoposide/carboplatin. For etoposide and carboplatin identical concentrations were used. Cell viability was assessed by MTT assay and is expressed as percentage of untreated cells. Data are shown as mean +/- SD of three independent experiments performed in triplicate.

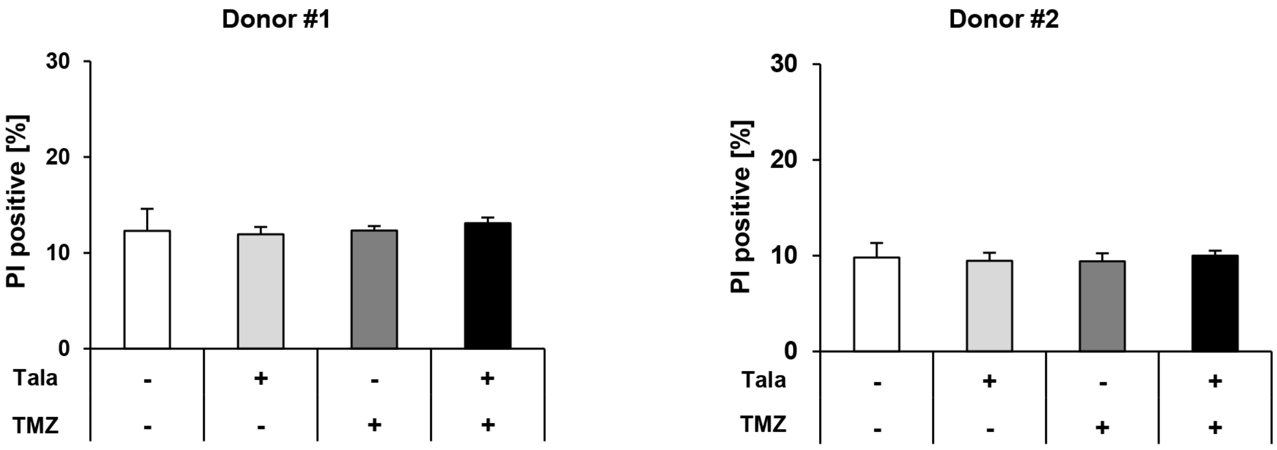

Supplementary Figure S2: Effect of PARP inhibitors and TMZ on peripheral blood lymphocytes. PBLs isolated from two independent donors were treated with 10 nM talazoparib and/or 100 µM TMZ for 48 hours and cell death was assessed by PI staining and flow cytometry to determine plasma membrane integrity. Data are shown as mean +/- SD of one independent experiment performed in triplicate.

Supplementary Table S1: Synergistic induction of apoptosis by combination treatment of PARP inhibitors and chemotherapeutics

Combination indices (CI) and fraction affected (FA) values were calculated as described in materials and methods for reduction of cell viability upon treatment with indicated concentrations of talazoparib and chemotherapeutics for 72 hours. Bold concentrations indicate combinations shown in Figure 3. Cut-off for antagonistic CI values was 2.

See Supplementary File 1
